# Albumin Substitution in Decompensated Liver Cirrhosis: Don’t Forget Zinc

**DOI:** 10.3390/nu13114011

**Published:** 2021-11-10

**Authors:** Kurt Grüngreiff, Thomas Gottstein, Dirk Reinhold, Claudia A. Blindauer

**Affiliations:** 1 Clinic of Gastroenterology, City Hospital Magdeburg GmbH, 39130 Magdeburg, Germany; thomas.gottstein@klinikum-magdeburg.de; 2 Medical Faculty, Institute of Molecular and Clinical Immunology, Otto-von-Guericke-University, 39120 Magdeburg, Germany; dirk.reinhold@med.ovgu.de; 3 Department of Chemistry, University of Warwick, Coventry CV4 7AL, UK; C.Blindauer@warwick.ac.uk

**Keywords:** liver cirrhosis, albumin, zinc

## Abstract

Decompensated liver cirrhosis has a dismal prognosis, with patients surviving on average for 2–4 years after the first diagnosis of ascites. Albumin is an important tool in the therapy of cirrhotic ascites. By virtue of its oncotic properties, it reduces the risk of cardiovascular dysfunction after paracentesis. Treatment with albumin also counteracts the development of hepatorenal syndrome and spontaneous bacterial peritonitis. More recently, the positive impact of long-term albumin supplementation in liver disease, based on its pleiotropic non-oncotic activities, has been recognized. These include transport of endo- and exogenous substances, anti-inflammatory, antioxidant and immunomodulatory activities, and stabilizing effects on the endothelium. Besides the growing recognition that effective albumin therapy requires adjustment of the plasma level to normal physiological values, the search for substances with adjuvant activities is becoming increasingly important. More than 75% of patients with decompensated liver cirrhosis do not only present with hypoalbuminemia but also with zinc deficiency. There is a close relationship between albumin and the essential trace element zinc. First and foremost, albumin is the main carrier of zinc in plasma, and is hence critical for systemic distribution of zinc. In this review, we discuss important functions of albumin in the context of metabolic, immunological, oxidative, transport, and distribution processes, alongside crucial functions and effects of zinc and their mutual dependencies. In particular, we focus on the major role of chronic inflammatory processes in pathogenesis and progression of liver cirrhosis and how albumin therapy and zinc supplementation may affect these processes.

## 1. Introduction

Human serum albumin is the most abundant protein in blood plasma, reaching concentrations of around 35–50 g L^−1^ in healthy conditions. The 65 kDa protein is synthesized in the liver and fulfils multiple functions. Albumin contributes up to 75% of the plasmatic oncotic pressure and has been shown to be a multifunctional protein with important binding capacity for metabolites, drugs, and metal ions and roles in the modulation of hemostasis and acid-base homeostasis, as well as possessing antioxidant, anti-inflammatory, and endothelium-stabilizing effects [1,2].

Owing to the reduced capacity of the liver for protein synthesis, patients with liver cirrhosis and ascites usually display reduced levels of serum albumin (hypoalbuminemia). In addition, liver cirrhosis not only decreases the levels of albumin but also leads to pronounced changes in its molecular integrity. These changes correlate with the severity of the cirrhosis and impair the multiple functions of albumin [3]. In patients with decompensated cirrhosis and patients with acute-on-chronic liver failure (ACLF), the binding efficiency of albumin for many of its ligands is significantly reduced [4]. As a result of quantitative and qualitative changes of albumin, the amount of “effective albumin” (eAlb) in the circulatory system can be dramatically reduced [5].

These changes affect the physiological actions of albumin in multiple ways. Since the 1940s, hypoalbuminemia has been known as a significant factor in the development of hepatogenic ascites [6,7], a condition associated with decompensated liver cirrhosis and later stages of liver disease. Ascites describes the accumulation of fluid in the abdominal cavity and is typically diagnosed by paracentesis, which is also a common treatment regimen for ascites. Albumin infusions have been standard after the therapy of hepatogenic ascites by large-volume paracentesis for over 25 years. The effectiveness of albumin in this condition relates to its major role in regulating plasma oncotic pressure; this is due to the fact that in healthy individuals, albumin makes up over half of plasma protein (oncotic effects).

Despite the recognized benefits of short-term albumin infusions to treat ascites, intravenous long-term treatments with albumin have gained significant importance only in the past decade, after the recognition of a range of non-oncotic effects of albumin. These effects include albumin’s scavenger function, its role in the maintenance of endothelial function, and more generally anti-inflammatory, antioxidant, and immunomodulatory effects [5,8].

Recent large-cohort studies have emphasized the importance of maintenance of albumin levels close to physiological values to ensure all major functions can be fulfilled. Indeed, according to very recent studies [9], maintenance of a serum level of 40 g L^−1^ stabilizes or even improves the course of disease in patients with liver cirrhosis with persistent ascites. The authors propose that long-term and personalized albumin therapy should aim to fill the gap between actual serum levels and this target value.

It may be suggested that the “actual serum levels” should take into account the molecular integrity of the remaining albumin—i.e., refer to fully functional albumin. Whilst “albumin binding function” has been proposed as one measure to assess the levels of effective albumin [5], at least one important physiological function of albumin often gets overlooked, namely its role as important physiological transporter of metal ions in the bloodstream. Although metal ions such as Ni^2+^, Co^2+^, and Cd^2+^ can bind in vivo, this is only of toxicological relevance [10]. In contrast, albumin binds and buffers several essential metal ions in blood plasma, including Ca^2+^, Mg^2+^, Cu^2+^, and Zn^2+^. This review focuses on Zn^2+^ because there is a clear correlation between zinc and albumin status in a range of conditions [11,12,13], and a well-founded understanding of structure, affinity, and allosteric effects on the major zinc-binding site of albumin [14,15,16]. Indeed, over 75% of patients with decompensated liver cirrhosis do not only present with hypoalbuminemia but also with a reduced serum zinc concentration [17,18] (see Section 5.4).

**Figure 1:**
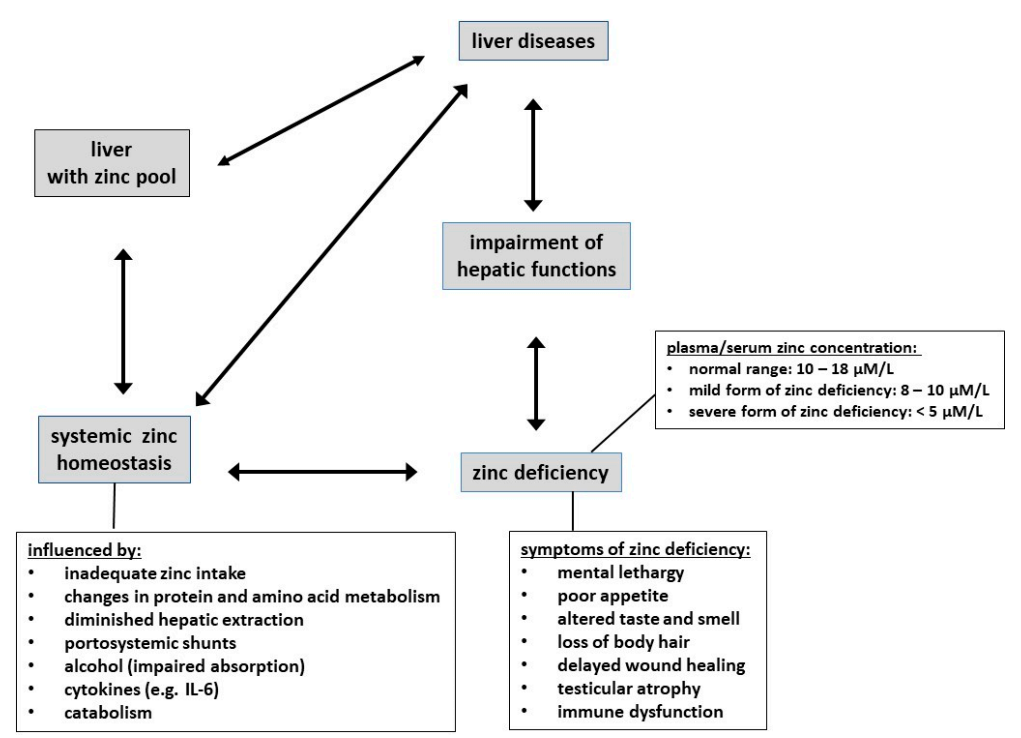
Schematic depiction of interactions between zinc homeostasis and the liver; factors influencing zinc in plasma/serum, symptoms of zinc deficiency and liver diseases.

Besides reviewing hallmarks of decompensated liver cirrhosis, we discuss alterations in albumin levels and molecular integrity and how this may affect the metabolism of the essential trace element zinc (see Figure 1). Particular focus is placed on details of the interactions between zinc and albumin and their impact on pathogenesis and therapy of liver cirrhosis.

## 2. Hallmarks of Decompensated Liver Cirrhosis

Along with continuous deterioration of functional parenchyma of the liver with subsequent reduction or abolition of its functions (metabolic decompensation), the progressive fibrotic process in liver cirrhosis is also accompanied by a total reorganization of its vascular tree (portal decompensation).

Acute decompensation is complex, and its development and progression remain unpredictable [19]. It can be precipitated by bacterial infection, liver injury, or other known and unknown mechanisms [6] and leads to the development of a range of complications, including ascites, hepatic encephalopathy (HE), hepatorenal syndrome (HRS), variceal bleeding, sarcopenia, and further bacterial infections [19,20,21]. In about a third of patients with acute decompensation, ACLF and multiple organ failure follow. One well-established factor in the development of decompensated cirrhosis is portal hypertension with splanchnic and systemic vasodilation. This accounts at least some degree for the development of hemodynamic abnormalities and cardiovascular dysfunction that are common in decompensated cirrhosis.

More recently, the central importance of systemic inflammation, with concomitant oxidative stress and mitochondrial dysfunction, has been recognized [6]. Subsequent metabolic changes lead to hemodynamic alterations, cardiovascular dysfunction, tissue damage and extrahepatic organ failure (kidney, heart, lungs, and brain), and severe impairment of the immune system. This impairment increases susceptibility to and severity of bacterial infections, which are also promoted by increased gut permeability, which can lead to translocation of bacteria or their components and endotoxemia. Bacterial products are sometimes referred to as pathogen-associated molecular patterns (PAMPs) and include lipopolysaccharides, peptidoglycans, flagellin, and nucleic acids from pathogens. Together with damage-associated molecular patterns (DAMPs, which arise from cell death and tissue damage, initially of the liver), these “danger molecules” are amongst the factors promoting the development of both localized and systemic inflammation [19].

### 2.1. Systemic Inflammation

Inflammation is a response of the innate immune system to molecular patterns associated with danger. Normally, inflammation is induced to deal with the pathogen or injury, and once this has been achieved, the inflammatory state is resolved. However, inflammation can become chronic and is a hallmark of many diseases including cirrhosis. Systemic inflammation can be recognized by increased numbers of leucocytes and increased C-reactive protein. Other biomarkers include both pro- and anti-inflammatory cytokines (TNF-α, IL-6, IL-8, IL-10, and IL-1 receptor antagonist). Their levels are associated with the severity of liver disease [19].

Many questions remain regarding the role of inflammation in disease progression in cirrhosis. In an interesting recent editorial entitled “Systemic inflammation and liver cirrhosis complications: driving or secondary event? How to square the circle?”, Samuel et al. [22] state that the involvement of systemic inflammation in ACLF is beyond doubt but that this is not the case for all complications of liver cirrhosis outside of this syndrome. Nonetheless, systemic inflammation certainly plays a significant role in the development of HE and kidney failure. In addition, both humoral and cellular inflammation, in conjunction with endothelial damage and activation, are key factors in the development not only of end-stage liver disease but also of extrahepatic complications of cirrhosis (e.g., cardiomyopathy) [23]. The involvement of systemic inflammation in the classical pathophysiological concept for ascites, the hepatorenal syndrome, and gastrointestinal bleeding are less clear. In Samuel’s view, there is a need for further immunological insights regarding cellular immunity (immunopathology), and he asks whether systemic inflammation is a primary or secondary event in liver disease [22].

Indeed, it is not always clear what induces systemic inflammation. In a number of cases, there may be involvement of bacterial infection, whilst translocation of bacterial products is thought to play a role in others [5], but in many other cases, the triggers cannot be identified.

Most recently, Costa et al. [24] confirm that systemic inflammation is a determining factor for progression of liver cirrhosis as well as being associated with decompensation.

### 2.2. Bacterial Infections

Infections increase mortality in cirrhosis four-fold [25] and have a poor prognosis, with 30% of patients dying within a month of infection and another 30% within a year [26]. The infections most frequently diagnosed are spontaneous bacterial peritonitis (SBP), urinary tract infections, pneumonia, and skin infections [27,28,29].

Findings of Wiest et al. [30,31] confirm and expand earlier studies that identified a decrease in the clearance function of the reticuloendothelial system (RES) of the liver, primarily represented by the Kupffer cells, macrophages that reside in the liver. This decreased efficiency of the RES allows intestinal bacteria and their products, e.g., endotoxins, to reach the systemic circulation. This endotoxemia impacts the pathogenesis of chronic liver disease [32,33]. In addition, in chronic liver disease, the intestinal venous blood can circumvent the liver’s RES, which disturbs antigen clearance irrespective of the actual functioning of the RES [34].

### 2.3. Hyperammonemia

Ammonia is a metabolite with considerable cell toxicity, and its plasma levels are usually regulated between 35–60 µmol L^−1^ [35]. The liver plays a central role in removal of ammonia from the bloodstream (by periportal hepatocytes: glutamine synthetase and the urea cycle). Due to portosystemic shunts (a consequence of reorganization of the hepatic vascular tree) and impaired liver function, ammonia is elevated in decompensated cirrhosis and ACLF (hyperammonemia) [36]. Hyperammonemia affects other organs, first and foremost the kidneys, which are also important for ammonia metabolism. Skeletal muscles can to some degree detoxify ammonia by the action of glutamine synthetase. However, cirrhotic patients with ascites often suffer from sarcopenia and hence reduced muscle mass, limiting their capacity for ammonia removal. Hyperammonemia leads to tissue damage and organ dysfunction and is a key factor in the development of HE. This is due to the increased production of glutamine by the action of glutamine synthetase in astrocytes; this leads to their swelling and edema in the brain [37]. Other organs and processes affected by hyperammonemia include the liver itself, the immune system, and skeletal muscle.

## 3. Albumin: Physiological Functions and Changes in Liver Disease

Albumin is synthesized predominantly in hepatocytes, where it is translated from a single gene as preproalbumin into the endoplasmic reticulum [2,5,38]. Processing of preproalbumin (609 amino acids for human serum albumin; HSA) leads to the secretion of the mature protein (585 amino acids) into the bloodstream. Here, serum albumin has a relatively long half-life of approximately 20 days [39]. Hepatocytes are not only crucial for albumin production but also its recycling in the liver. Therefore, a cirrhotic liver is less able to maintain physiological albumin levels.

The synthesis of albumin is regulated by several factors, with its plasma concentration being the most important one. Hypoalbuminemia stimulates synthesis, whereas albumin infusion suppresses it [6]. In patients with decompensated liver cirrhosis, albumin metabolism is disturbed by a range of factors. Besides diet and the consumption of alcohol or other toxic substances, a major role is played by chronic inflammation of the liver, which is mediated by pro-inflammatory cytokines (IL-6, TNF-α). Thus, inflammation of the liver decreases plasma albumin levels, and low plasma albumin is therefore a biomarker for systemic inflammation. However, albumin levels are also governed by body mass and are hence a general marker of nutritional status [40]. In addition, the physiological changes that are associated with inflammation also impact the molecular integrity of albumin, with pronounced effects on its functions.

Besides being important for maintaining appropriate oncotic pressure, albumin exerts many non-oncotic effects, most of which are related to its ability to bind a plethora of diverse substances, including drugs, toxins, and antigens. In addition, albumin represents the largest contingent of antioxidant thiols in the bloodstream, enabling it to counteract oxidative stress.

Perhaps even more important are albumin’s functions as a transporter of essential metabolites, first and foremost free (=non-esterified) fatty acids (FFAs) and zinc. There are five well-described medium-to-high-affinity FFA-binding sites, plus at least two more low-affinity sites on albumin [41]. Section 5.3 gives further details on how elevated plasma FFAs affect albumin’s zinc-binding ability.

Only reduced native albumin (sometimes referred to as “effective albumin”; eAlb) is capable of fulfilling all functions described so far [5]. Many of the physiological alterations encountered in liver disease, including systemic inflammation, oxidative stress, elevated blood glucose, and elevated plasma fatty acids, compromise molecular integrity and/or binding capacity of albumin. Therefore, patients with liver disease do not only suffer from hypoalbuminemia, but a larger proportion of their remaining albumin is dysfunctional. Indeed, correlations between albumin’s decreased functional capacity and mortality in patients suffering from decompensated cirrhosis have been established [42], with the level of damaged albumin being a good predictor of disease progression. Conversely, recent studies have suggested that replenishing serum albumin levels by long-term infusions with albumin may improve patient outcomes.

## 4. Albumin Substitution in Decompensated Liver Cirrhosis: Latest Insights

Currently, treatment with intravenous albumin is indicated for the prevention of circulatory dysfunction after large-volume paracentesis for ascites removal (using plasma expansion to counter hypovolemia), the prevention and management of the HRS, and in SBP [6]. Treatment regimens have many variables, including dosage and duration. The main rationale for long-term albumin substitution has, until recently, been the improvement of circulatory functions, which may prevent a range of acute complications of cirrhosis [6]. Research into albumin supplementation has recently been reinvigorated after discovery of its immunomodulatory and anti-inflammatory properties.

However, large-cohort clinical data on treatment with albumin in decompensated cirrhosis are still scarce and somewhat ambiguous. No benefits of albumin supplementation were discerned in the “Midodrine and Albumin for Cirrhotic Patients in the Waiting List for Liver Transplantation” (MACHT) [43] or the “Albumin To Prevent Infection in Chronic Liver Failure” (ATTIRE) [44] randomized controlled trials (RCT). In contrast, the “Albumin for the Treatment of Ascites in Patients with Hepatic Cirrhosis” (ANSWER) [45] RCT and the smaller Pilot-PRECIOSA and INFECIR-2 studies have reported various benefits, most crucially for overall patient outcomes.

The results from the ANSWER study have proven that intravenous treatment with human albumin improves the course of disease, with a lower incidence of almost all common complications and longer survival of patients with decompensated liver cirrhosis and pronounced ascites [45]. Crucially, the dosages in [45] were considerably higher than those in the MACHT and ATTIRE studies and aimed at increasing serum albumin levels to a median level close to 40 g L^−1^, whilst no increases were achieved in the MACHT study, and the target value in the ATTIRE study was 30 g L^−1^. From these (limited) data, it would appear that only restoration of physiological levels of functional albumin provides tangible benefits. In the light of the discussion in Section 3, the molecular integrity of the patient’s own albumin and that of the commercial albumin preparation should also be monitored.

### Immunomodulatory and Anti-Inflammatory Effects

The modes of action of albumin to modulate immune responses and reduce systemic inflammation are likely multifactorial and have only begun to be unraveled. The most obvious ways in which albumin may affect processes involved in immune response and inflammation is by binding, at least temporarily, one of the interaction partners, typically a small molecule. These include toxic metabolites (bilirubin, biliary acids), inflammatory mediators (including both pro- and anti-inflammatory eicosanoids [46]), reactive oxygen species, and at least some bacterial products [6]. The lack of effective albumin may thus promote the spread of bacterial products, but other pathways are emerging.

A study by Casulleras et al. [47] on leukocytes isolated from patients with ACLF demonstrated that HSA can inhibit the expression and release of cytokines in response to challenge with bacterial DNA. These effects are independent of oncotic and scavenger functions and could be reproduced by incubation of the leukocytes with recombinant HSA. It is suggested that albumin is taken up by receptor-mediated endocytosis into leukocytes—as also observed for hepatocytes and endothelial cells [47]. It is thought that the immunomodulatory function may be related to affecting the interaction of PAMPs with toll-like receptor (TLR) signaling. The authors stress that HSA did not compromise leukocyte defensive mechanisms and that HSA thus did not lead to immunosuppression [48].

Immunomodulatory effects, reduction in systemic inflammation and cardiocirculatory dysfunction were observed in an evaluation of the Pilot-PRECIOSA and INFECIR-2 studies [8]. Furthermore, albumin administration has also been suggested to help reverse endotoxemia, hyperammonemia, and hyponatremia [5].

## 5. Zinc: Physiological Functions, Deficiency, Homeostasis, and Changes in Liver Disease

### 5.1. Physiological Functions

Zinc is an essential trace element in human health and plays a fundamental role in metabolic, immunological, and many other biological processes. At least 10% of the human proteome requires zinc for its function [49]; hence it is fair to say that zinc is involved in all major physiological processes. In enzymes, catalytic zinc accelerates biochemical reactions by means of its direct participation in substrate binding and turnover and by stabilizing protein structure. For example, without zinc, the synthesis of DNA, RNA, and proteins is impossible [50,51]. Structurally, zinc is involved in transcription factors, many of which are so-called zinc fingers. In addition, more recent work has shown that many effects of zinc are based on the intra- and extracellular regulatory function of the zinc ion (Zn^2+^), via transient interactions with proteins [52]. Zinc thus exercises numerous and varied regulatory functions with respect to gene expression, hormones, and their receptors. Its roles in energy metabolism are particularly noteworthy; adequate zinc homeostasis is required for normal insulin synthesis and storage, as this involves a zinc-bound hexameric assembly. In blood plasma, zinc is an important factor regulating blood clotting [53].

Moreover, zinc serves as a membrane stabilizer and is also important for mucosal health, e.g., in the intestines [54]. The authors of [54] discuss molecular and genetic regulation of numerous processes in various organs in the context of diarrhea, zinc deficiency, and zinc supplementation. In response to mitogens, zinc-deficient individuals displayed reduced proliferation of lymphocytes and other alterations, all of which could be rectified by zinc supplementation. In a recent study, Sarkar et al. demonstrate impressively how zinc affects the integrity of the intestinal epithelial barrier and inflammatory responses during bacterial infection [55]. In zinc-deficient mice infected with Shigella, intestinal permeability was severely disturbed, leading to bacterial colonization, translocation, and shedding. The authors view these findings as confirmation for the role of zinc deficiency in development and severity of bacterial infections and inflammation.

Zinc is also important for sensory functions and is essential for the functioning of the immune system [51,56]. As zinc deficiency results in altered numbers and dysfunction of all immune cells [57], individuals with decreased zinc levels have an increased risk for infectious diseases, autoimmune disorders, and cancer [57,58,59]. On the cellular and organismal level, zinc has been shown to have antioxidant, anti-inflammatory, immunomodulatory, and antiapoptotic effects [60].

Finally, it is worth mentioning that although zinc is generally not considered to be toxic to man, the Zn^2+^ ion is toxic to cells at concentrations as low as a few nanomolar [61]. This emphasizes the importance of ensuring that proteins involved in zinc homeostasis—including albumin—are present and functional.

### 5.2. Zinc Deficiency: Prevalence and Symptoms

Zinc deficiency [62], the systemic lack of zinc required to maintain the functions described has considerably higher prevalence than commonly recognized. To some degree, this lack of appreciation of this condition is due to the fact that there are no specific biomarkers of zinc status similar to ferritin and transferrin in iron deficiency [63]. This “elusiveness” is in fact based on its many and diverse metabolic effects and its interactions with a plethora of proteins.

In the absence of defined biomarkers for zinc deficiency, the determination of zinc concentrations in serum or plasma with defined trace element-free collection systems is often considered as the most appropriate measure that can be easily reproduced in everyday clinical practice. Indeed, in official healthcare policy documents, the term “zinc deficiency” describes a reduction in the zinc levels in serum or plasma with corresponding clinical symptoms. Measurement in either of these two compartments is the only indicator recommended by the World Health Organization (WHO), UNICEF, and other organizations for estimating the zinc status in the population [64]. The Biomarkers of Nutrition for Development (BOND) Zinc Expert Panel also recommends plasma zinc concentrations as the main biomarker [65].

However, plasma/serum zinc levels are on the one hand well-regulated within a relatively narrow range (10–18 μM [66]) and on the other hand are influenced by many factors [67] and hence in fact not a reliable indicator of zinc status. Indeed, when zinc intake is reduced, there is a reduction in endogenous losses to conserve zinc [68], and zinc is mobilized from small, rapidly exchangeable pools in liver and possibly bone [63,69,70,71], whilst a large number of studies have shown that plasma levels do not change substantially in dependence on zinc intake [67]. Therefore, plasma levels are, normally, only depressed in more severe cases of zinc deficiency, i.e., in patients where all body stores are completely depleted. It may be noted that milder forms of zinc deficiency, i.e., some depletion of whole-body zinc, but with no manifestation in plasma/serum levels, may still have adverse effects. However, since such a state is currently difficult to diagnose, zinc deficiency is in all likelihood vastly more widespread than commonly recognized. Symptoms of moderate to pronounced zinc deficiency include mental lethargy, poor appetite, altered smell and taste, loss of body hair, delayed wound healing, testicular atrophy, immune dysfunction, and diminished drug elimination capacity [62,72]. Moreover, since zinc is involved in virtually all major biochemical and cellular processes, its deficiency is associated with impairment of numerous metabolic processes, including disturbed energy metabolism due to impairment of insulin production, secretion, and storage, and reduced resistance to infections due to impaired immune functions.

Finally, there is a very close relationship between zinc deficiency and cellular oxidative stress. Cell-damaging oxidative stress as a consequence of zinc deficiency is a fundamental principle [52]. In turn, cellular stress (altered or disturbed biological processes and their consequences in the cell) may cause a loss of zinc. The subsequent cellular zinc deficiency not only increases oxidative stress but also leads to endoplasmic reticulum stress. The result is a vicious circle [52].

Importantly, zinc deficiency is not only caused by insufficient intake or malabsorption but also occurs in chronic disorders of the heart, liver, pancreas, kidney, diabetes mellitus, obesity, rheumatoid arthritis, and more [62,73,74]. Many if not all of these disorders are also associated with systemic inflammation. It is also noteworthy that zinc deficiency becomes more prevalent with age, which is thought to account at least in part for reduced immune function in the elderly [75].

### 5.3. The Central Role of Albumin in Zinc Homeostasis

#### 5.3.1. General Considerations

Almost 95% of body zinc is located intracellularly. The regulation of cellular zinc homeostasis takes place via a complex network of metal transporters [76] and buffering systems (including metallothioneins) that react to changes in the availability of zinc in nutrition, chronic diseases, infections and many other processes [73]. Although circulatory zinc (predominantly plasma zinc) only makes up ca. 0.1% of total body zinc, it is critical in whole-body distribution [16,67]. Plasma/serum zinc turns over 150 times per day [67]; this gives an impression of the dynamic nature of zinc metabolism and its importance for all major physiological processes.

In plasma, zinc is mainly bound to albumin (approximately 70%) and α-2-macroglobulin (ca. 10–20%) [69,77]. Zinc in the latter protein is firmly bound and non-exchangeable. In contrast, albumin-bound zinc is the major component (>90%) of the exchangeable Zn^2+^ pool in plasma [69]. Dissociation constants for Zn^2+^-albumin complexes are in the high nanomolar to low micromolar range [77,78]. This moderate binding affinity for Zn^2+^ is optimized on the one hand to minimize cytotoxic effects of Zn^2+^ and on the other to permit access to Zn^2+^ for other proteins. This includes other plasma proteins (e.g., histidine-rich glycoprotein and other proteins involved in coagulation [15,79]) and zinc transporters on the membranes of cells in direct contact with blood plasma (erythrocytes, leukocytes, platelets, endothelial cells, etc.). Another way to look at this is to consider albumin as the major determinant of plasma zinc speciation, with (effective) albumin concentration governing zinc availability to proteins and cells [80,81]. These studies and more recent work have started to explore how albumin affects the spatial and temporal distribution of zinc and implications of this dynamics [79].

Typically, HSA/Zn ratios are, under normal conditions, in the order of 30:1 [82]. Consequently, and since albumin is the major zinc-binding protein in plasma, alterations in its levels are often accompanied by similar alterations of plasma zinc. This is indeed also the case for diseases that are accompanied by chronic inflammation [40].

#### 5.3.2. Molecular Details of Zinc-Albumin Interactions

**Figure 2:**
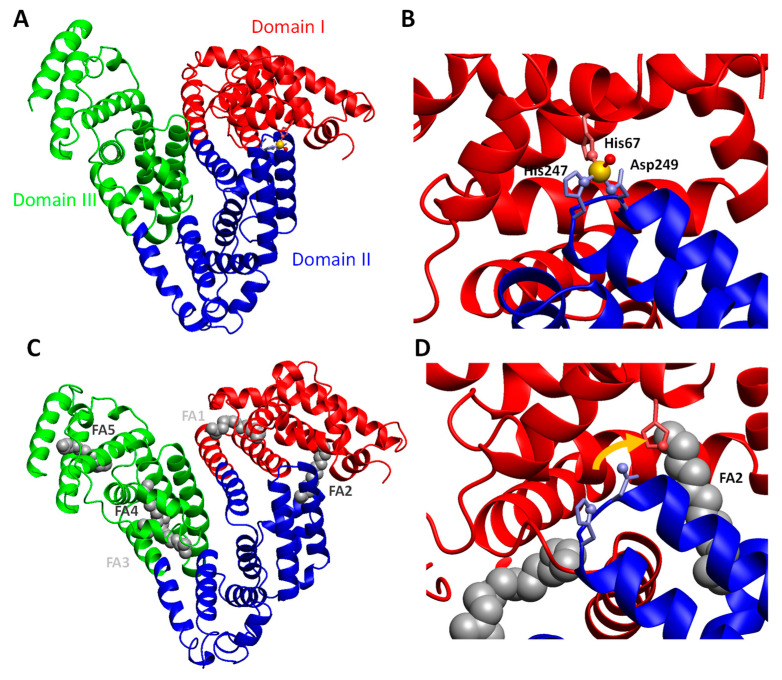
Structural aspects of zinc and FFA binding to HSA. (**A**) Domain structure and location of the major zinc binding site (golden sphere) on human serum albumin (pdb 5ijf; [83]). (**B**) Zinc is bound by three amino acid residues as indicated, with water as a fourth ligand. (**C**) Location of the five major FFA-binding sites on HSA as observed in presence of myristate (pdb 1bj5; [84]). FA2, 4 and 5 are high-affinity sites, whilst FA1 and FA3 are medium-affinity sites. Two further low-affinity sites are not occupied in this structure. (**D**) Disruption of the major zinc binding site by FFA binding to site FA2. His67 in domain I moves (as indicated by the orange arrow) by ca. 6 Å relative to His247 and Asp249 in domain II (see [14,15]).

Albumin is a protein composed of three homologous domains (Figure 2A). The major zinc-binding site on albumin is formed by three residues: His67 from domain I and His247 and Asp249 from domain II (Figure 2B; [83]). Thus, this site is located at the interface of two domains. Importantly, there are few other interactions between those domains, and their mutual orientation is subject to conformational changes, depending on bound ligands. Most prominently, the binding of free fatty acids (FFAs) in binding site FA2 elicits a change in this domain interface (Figure 2C; [84]) that disrupts the zinc binding site (Figure 2D; [14,85]. This leads to a dramatic decrease in the zinc-binding capacity for both bovine and human albumin [14,15]—a classical case of allostery.

Consequently, the Zn^2+^ that can no longer bind to albumin binds increasingly to other plasma proteins, as demonstrated recently by Coverdale et al. [81]. Importantly, the levels of FFA in plasma vary as a consequence of both physiological (e.g., fasting, stress, extreme exercise) and pathophysiological (obesity, diabetes, liver disease) conditions [16]. It follows that plasma zinc and whole-body distribution of zinc are disturbed in diseases that are associated with chronically increased levels of plasma FFAs. The consequences of this allosteric interaction between FFAs and zinc have only just started to be investigated. A recent study by Sobczak et al. [79] has shown that such FFA-induced alterations of zinc speciation may be involved in disturbed blood clotting in type 2 diabetes patients.

The allosteric mechanism described for FFAs and Zn^2+^ also plays a role in the clinical albumin–cobalt binding (ACB) test [86,87]. This test was developed to detect a biomarker called “ischemia-modified albumin” (IMA) [88]. Various hypotheses have been put forward and tested to identify the modifications that IMA has undergone. The only hypothesis that explains all clinical observations, including a rapid (within hours) decline of IMA after resolution of an ischemic event, is that IMA corresponds to normal albumin with increased FFA loading. A correlation between IMA and FFA levels has also been reported [89], and crucially, decreased Co^2+^-binding capacity of FFA-loaded BSA has been demonstrated in vitro [86]. This molecular mechanism also explains why a negative ACB test is not just a reliable method to rule out ischemia as a cause of chest pain but also why “IMA” is elevated in a plethora of other conditions that are not associated with ischemia but characterized by elevated plasma FFAs [87].

Most importantly, plasma FFAs are increased in decompensated cirrhosis [90]. Correspondingly, IMA, expressed as an “ischemia-modified albumin ratio” (IMAR), has been found to correlate with disease severity in such patients [42]. Indeed, these authors also noted a negative correlation between IMAR and FFA-binding ability. Here, we propose that these increased FFA levels further compromise the zinc binding ability of albumin and hence also affect whole-body zinc supply.

Further deterioration of zinc transport and distribution by albumin may be caused by glycation [91], another common molecular alteration encountered in liver disease.

### 5.4. Zinc, the Liver, and Changes in Plasma Zinc of Patients with Cirrhosis

The liver is essential for zinc homeostasis, and in turn, zinc deficiency leads to the impairment of many hepatic functions. Accordingly, liver diseases can alter zinc levels and in turn may be influenced by zinc deficiency [72,92].

The liver plays an important role in the metabolism of zinc and other trace elements [93]. The liver zinc pool exchanges fast, owing to the actions of multiple ZIP and ZnT transporters that are regulated by a range of factors including a variety of hormones (insulin, glucagon, glucocorticoids; [94,95,96]). Depending on the metabolic situation, these agents trigger changes in zinc metabolism. For example, elevated plasma glucose after a meal correlates with depressed plasma zinc [97]; this is thought to be mediated in part by liver ZIP14 [98]. In turn, plasma zinc levels (and speciation) is also thought to affect insulin activation and clearance by the liver: the insulin form secreted by the pancreas is an inactive hexamer stabilized by two zinc ions. The removal of these zinc ions by dilution and by complexation by other proteins such as albumin promotes the formation of an active monomer, which is also the form that is cleared by the liver [99]. Thus, the liver mediates crosstalk between zinc and metabolic hormones in multiple ways and directions.

Stress or mediator substances, such as proinflammatory cytokines and lipopolysaccharides, can also affect plasma zinc levels. For example, upregulation of ZIP14 in hepatocytes by pro-inflammatory IL-6 plays a major role in the hypozincemia (i.e., decreased plasma levels of zinc) that accompanies the acute-phase-response [100] and is now recognized as an aspect of “nutritional immunity”.

Changes in zinc status directly influence gene expression. Systemic zinc deficiency affects different hepatic functions and, because of the liver’s central role in metabolism, especially the carbohydrate, lipid, and protein metabolism [17,101,102,103].

Zinc deficiency is common in patients with liver cirrhosis [12,17,18]. Here, the deficiency is manifested both in reduced serum zinc and in patients often displaying the classical symptoms of zinc deficiency described in Section 5.2. The deficiency is caused by a variety of factors, such as inadequate intake, changes in protein and amino acid metabolism (including hypoalbuminemia), portosystemic shunts, impaired absorption, and the effects of bacterial endotoxins and cytokines, mainly IL-6 [13,17,104]. In addition, sarcopenia can lead to a substantial loss of body zinc in the urine [105]. Furthermore, the diuretic therapy in these patients leads not only to an increased renal zinc excretion but also to reduced serum albumin and hence additionally reduced capacity to bind zinc in plasma [17,106]. This means that the diuretic therapy exacerbates some complications of liver disease; this should be compensated for.

The multifactorial causes for zinc deficiency coalesce in the central hub of zinc metabolism, i.e., plasma and albumin. As explained previously, albumin substitution is becoming more common; we suggest that albumin substitution therapies might benefit from being complemented with zinc supplementation.

## 6. Zinc Supplementation in Liver Disease

Results of studies on zinc supplementation in patients with liver cirrhosis are ambiguous. Recent meta-analyses [107,108] revealed only marginal benefits of zinc supplementation for the clinical course of cirrhosis. These conclusions are contrasted by many smaller studies where positive influences were discerned for metabolic disorders that are caused by zinc deficiency. This includes reductions in ammonia levels, improvement of glucose tolerance, decrease in insulin resistance, stimulation of liver regeneration [109,110], and several hepatoprotective effects, e.g., induction of metallothionein synthesis; improvement of protein synthesis in the liver; inhibition of lipid peroxidation; anti-oxidative effects against cellular, mitochondrial, and ER stress; and anti-inflammatory effects [111]. In a recent study on the effects of a 3-month course of zinc supplementation (30 mg elemental Zn) on metabolic profile and oxidative stress in obese patients with non-alcoholic fatty liver disease (NAFLD), Fathi et al. [112] demonstrated improvement of insulin resistance and oxidative stress. Lipid profiles and weight were not affected. Vilar Gomez et al. [113] and Takuma et al. [114] observe that zinc supplementation may counteract the increased permeability of the gut and bacterial spillover into the systemic circulation in liver cirrhosis.

The efficacy of zinc supplementation is also controversial in relation to HE [48]. Studies that demonstrate a positive influence on HE [107,114,115,116,117,118,119] are contrasted by others in which no effect was found [118]. An early double-blind study of 22 cirrhotic patients treated with zinc acetate (600 mg/d for 7 days) [115] found—besides normalization of serum zinc concentrations—an increase in the rate of urea production and improvement in performance of psychometric tests in patients treated with the zinc preparation. Other authors report on improved night vision and improvements in HE stage in patients receiving zinc histidine [116]. In addition, Van der Rijt et al. [117] have reported an association between episodes of overt HE and zinc deficiency, with improvement in the patient’s HE stage following zinc supplementation. Horiguchi et al. [118] and Miwa et al. [119] also report positive effects of zinc supplementation in HE and the overall condition of patients with cirrhosis and confirmed zinc deficiency. In contrast to these positive effects of zinc supplementation on HE, Riggio et al. [120] did not observe a corresponding improvement in a placebo-controlled study that followed patients for seven days.

Our own experience during a long-term therapeutic observation over 42 months has shown that long-term treatment with zinc histidine or zinc aspartate results in normalization of serum zinc and ammonia concentrations and reduction in HE stage in 55–60% of patients with HE as a complication of liver cirrhosis [111]. These parameters were examined in each patient before therapy, 3 and 6 months after start of therapy, and then every 6 months up to 42 months. The administration of zinc was subject to regular monitoring of serum zinc levels (every 6 to 8 weeks) and was discontinued upon normalization of serum zinc concentration. Resumption of zinc supplementation after the decrease in zinc levels effected an increase in zinc and a decrease in ammonia levels in most patients. This episodic behavior was noted over the whole observation time.

These examples demonstrate that further well-designed studies are required, with clear directives regarding study design, which observations are selected, the duration of zinc supplementation, which formulation is chosen, and many more factors.

### 6.1. What Are the Conceivable Effects of Zinc Supplementation in HE?

Although the pathogenesis of HE is multifactorial, high levels of ammonia (hyperammonemia) play a key role. Ammonia induces swelling of the astrocytes, and associated changes disrupt neuronal energy production in the brain. This edema of the astrocytes causes oxidative and nitrosative stress [121]. Interestingly, the latter causes not only oxidation of messenger RNA, with resulting disturbance of postsynaptic protein synthesis and effects of learning and memory processes, but also an elevation of free intracellular Zn^2+^ [122]. The latter effect is due to the oxidation of protein thiols that are involved in zinc binding.

Other factors beyond ammonia that are involved in the pathogenesis of HE include infections, the effects of proinflammatory cytokines, and neutrophils [121,123]. Experimental data [72,124,125,126] suggest that the efficacy of zinc supplementation in HE depends on its promotion of the effects of zinc-dependent enzymes in the urea cycle (ornithine carbamoyltransferase; liver) and/or the glutamine cycle (glutamine synthetase; liver and muscle). This contributes to normalization of hyperammonemia. In addition, zinc supplementation in patients with liver cirrhosis improves liver function by stimulating metabolic processes in the hepatocytes [126].

### 6.2. Practical Recommendations

Based on current knowledge on the roles of zinc in biological processes in general, and on zinc deficiency as potential pathogenic cofactor in a range of chronic diseases in particular, the serum or plasma zinc concentration should be measured when typical symptoms of zinc deficiency are detected.

In patients with liver cirrhosis or diabetes mellitus, measurement of serum/plasma zinc concentration (ZC) is recommended even before such symptoms arise, as in these conditions, ZC is often already decreased [48]. If decreased ZC is reproducibly demonstrated, zinc supplementation is medically indicated. This treatment should occur in a controlled manner, with regular tests every 6 to 8 weeks. According to our own experiences over many years, the dosage of the zinc supplement should be adapted to compensate for the reduction in ZC [50]. The reference range for serum zinc is 11–23 µmol L^−1^ or 60–120 µg dL^−1^. Table 1 shows recommended doses, expressed in elemental zinc. Based on our own experiences, Zn-histidine or Zn-aspartate are best absorbed in patients suffering from chronic liver diseases [50]. It is recommended that the zinc supplement is taken 1 h before or after meals; this prevents a reduction in zinc absorption in the gut caused by complexation by phytate or other components of plant-based food. Further details can be found in reference [50].

Once ZC has reached normal levels, supplementation can be paused, but further tests are advisable. If ZC falls once more, zinc supplementation should be resumed. Apart from the severity of the liver cirrhosis (ascites, HE, infections), additional factors such as quality and form of diet, possible alcohol consumption, comorbidities (e.g., diabetes mellitus), and medication (e.g., diuretica) are important factors affecting the rate at which normalization of ZC is achieved. Stable replenishment of zinc stores in bone, liver, and muscles can take up to 6 months [50].

The maximal doses for zinc supplementation are at present not clearly defined but are of relevance in case of long-term administration [50]. For instance, dosages of 100 mg or more elemental zinc per day can cause severe immunological damage [127,128]. Experimental studies showed that zinc concentrations higher than 0.5 mM (equivalent to a daily dose of 45 mg elemental zinc) lead to toxic effects on immune cells with inhibition of DNA synthesis and cytokine production [129]. Conversely, according to Prasad [130], oral zinc up to 45 mg/day is not considered toxic.

A recent letter by Nath et al. [131] in the “*Indian Journal of Critical Care Medicine*” emphasizes clearly the importance of controlled zinc supplementation. The authors report an increase in fungal infections (mucomycosis) in patients with SARS-CoV2 infection that had taken uncontrolled high doses of zinc. Zinc is essential for growth of various pathogenic fungi [132]. It affects several pathogenic mechanisms by directly influencing fungal proteins that promote infection in mammals [133]. Accordingly, the authors warn of “rampant usage” of zinc supplements without medical oversight.

## 7. Conclusions

**Figure 3:**
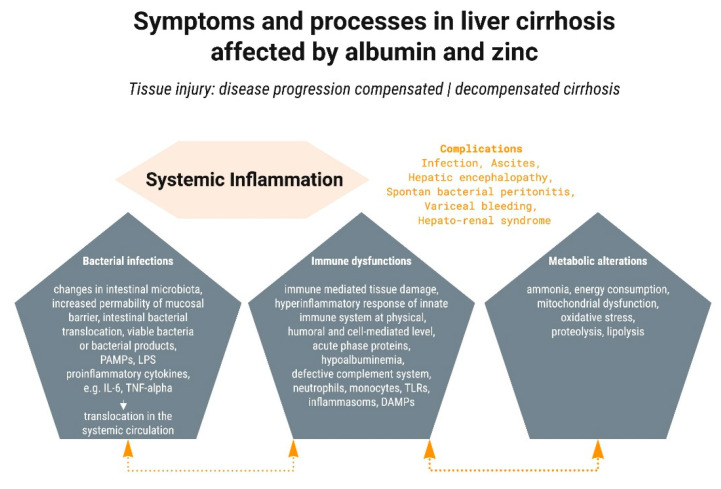
Schematic presentation of the influence of albumin and zinc on the pathogenesis of liver cirrhosis. Arrows indicate the relations and the interactions between bacterial infections, immune dysfunctions, and metabolic alterations.

Chronic inflammation is often the driving force in the development and progression of chronic diseases, including those of the liver. The spread to other organs and the dysfunction of metabolic, immunological, hemostatic, cerebral, and other processes and functions depend on severity and of course the genesis (viral, alcoholic/non-alcoholic fatty liver, autoimmune, chronically cholestatic, metabolic) of the disease. In these complex scenarios, both albumin (due to both oncotic and non-oncotic effects) and zinc (due to its equally pleiotropic effects) occupy central positions (Figure 3). There are mutual interactions and dependencies between the liver, albumin, and zinc. Patients with decompensated liver cirrhosis have ca. 75% decreased albumin and plasma zinc levels. This means that in these patients, the majority of essential biological functions of these agents can only be fulfilled in a limited way, or not at all.

With current knowledge regarding the importance of zinc for biological processes in general (Section 5.1), and the role of zinc deficiency as a decisive factor in the pathogenesis of many chronic diseases in particular (Section 5.2), it should be clear that in the presence of typical symptoms of zinc deficiency and depressed plasma zinc levels, controlled zinc supplementation should be administered.

Studies on the impact of zinc on liver cirrhosis (genesis, stages, ascites, HE, HCC, bacterial infections) are extremely heterogeneous in design, patient selection, complications, medication, formulation of the zinc supplement, dosage, duration of treatment, timing of lab tests, etc. Thus, it has thus far not been possible to define a generally accepted recommendation.

Zinc fulfils many of the characteristics elaborated by Caraceni et al. [9] for “disease-modifying agents” in decompensated liver cirrhosis. Similar to suggestions by these authors regarding the efficacy of albumin, simvastatin, rifampicin, and other hard-to-resorb antibiotics, supplementation with zinc should be evaluated in large-cohort, well-designed, high-quality randomized controlled trials and/or observational studies.

## Figures and Tables

**Table 1:** Recommended dosages of zinc supplements for different degrees of zinc deficiency confirmed by decreased serum zinc concentration (ZC).

ZC (µmol L^−1^)	Recommended Regime (Elemental Zn, Daily over 6 Weeks)
11–9.5	10–15 mg
below 9.0	30 mg
below 6.0	up to 45 mg
